# Management of multimorbidity using a patient-centred care model: a pragmatic cluster-randomised trial of the 3D approach

**DOI:** 10.1016/S0140-6736(18)31308-4

**Published:** 2018-07-07

**Authors:** Chris Salisbury, Mei-See Man, Peter Bower, Bruce Guthrie, Katherine Chaplin, Daisy M Gaunt, Sara Brookes, Bridie Fitzpatrick, Caroline Gardner, Sandra Hollinghurst, Victoria Lee, John McLeod, Cindy Mann, Keith R Moffat, Stewart W Mercer

**Affiliations:** a Centre for Academic Primary Care, NIHR School for Primary Care Research, Population Health Sciences, Bristol Medical School, University of Bristol, Bristol, UK; b Bristol Randomised Trials Collaboration, Population Health Sciences, Bristol Medical School, University of Bristol, Bristol, UK; c NIHR School for Primary Care Research, Centre for Primary Care, Division of Population of Health, Health Services Research and Primary Care, Manchester Academic Health Science Centre, University of Manchester, UK; d Population Health Sciences Division, School of Medicine, University of Dundee, Dundee, UK; e Institute of Health and Wellbeing, University of Glasgow, Glasgow, UK

## Abstract

**Background:**

The management of people with multiple chronic conditions challenges health-care systems designed around single conditions. There is international consensus that care for multimorbidity should be patient-centred, focus on quality of life, and promote self-management towards agreed goals. However, there is little evidence about the effectiveness of this approach. Our hypothesis was that the patient-centred, so-called 3D approach (based on dimensions of health, depression, and drugs) for patients with multimorbidity would improve their health-related quality of life, which is the ultimate aim of the 3D intervention.

**Methods:**

We did this pragmatic cluster-randomised trial in general practices in England and Scotland. Practices were randomly allocated to continue usual care (17 practices) or to provide 6-monthly comprehensive 3D reviews, incorporating patient-centred strategies that reflected international consensus on best care (16 practices). Randomisation was computer-generated, stratified by area, and minimised by practice deprivation and list size. Adults with three or more chronic conditions were recruited. The primary outcome was quality of life (assessed with EQ-5D-5L) after 15 months' follow-up. Participants were not masked to group assignment, but analysis of outcomes was blinded. We analysed the primary outcome in the intention-to-treat population, with missing data being multiply imputed. This trial is registered as an International Standard Randomised Controlled Trial, number ISRCTN06180958.

**Findings:**

Between May 20, 2015, and Dec 31, 2015, we recruited 1546 patients from 33 practices and randomly assigned them to receive the intervention (n=797) or usual care (n=749). In our intention-to-treat analysis, there was no difference between trial groups in the primary outcome of quality of life (adjusted difference in mean EQ-5D-5L 0·00, 95% CI −0·02 to 0·02; p=0·93). 78 patients died, and the deaths were not considered as related to the intervention.

**Interpretation:**

To our knowledge, this trial is the largest investigation of the international consensus about optimal management of multimorbidity. The 3D intervention did not improve patients' quality of life.

**Funding:**

National Institute for Health Research.

## Introduction

There is increasing awareness of the importance of multimorbidity, defined as patients living with two or more chronic health conditions. One in four people in the UK and the USA have multimorbidity, increasing to at least two-thirds of those older than 65 years.^1^, ^2^ Multimorbidity is associated with reduced quality of life, impaired functional status, worse physical and mental health, and increased mortality.^3^ The increasing prevalence of multimorbidity, driven by the ageing population, represents a major challenge to all health-care systems because these patients are heavy users of services. In the USA, people with multimorbidity account for more than two-thirds of total health spending.^2^

Efforts to improve the care of patients with chronic diseases have focused on developing guidelines to implement standardised care for each disease. However, this approach can have disadvantages for patients with multimorbidity.^4^ Recommendations based on disease-specific guidelines can be inappropriate for patients with co-existing conditions.^3^ If each condition is considered in isolation, patients can be prescribed numerous drugs and lifestyle changes, and are expected to attend frequent health-care appointments. Therefore, treatment itself can represent an excessive burden for patients with multimorbidity, alongside their burden of illness.^5^ Furthermore, segmentation of care by disease means that health care for these patients is often fragmented and poorly coordinated. Older adults describe wanting one professional to take continuing responsibility for their overall care, and to consider their personal situation and preferences when advising about treatment decisions.^6^

Recognising these problems, organisations in England,^3^ the USA,^2^, ^7^ Europe,^8^ and internationally^9^ have published guidance about improving the management of patients with multimorbidity, and the US Department of Health and Human Services has called for a paradigm shift in how care is provided.^2^ There is broad consensus about the key components of such an approach, which reflect a patient-centred care model^10^ and insights from the Chronic Care Model.^11^ These components include a regular comprehensive review of patients' problems according to their individual circumstances, a focus on quality of life and function as well as disease control, tailoring treatment recommendations to each individual's priorities and situation, balancing risks and benefits of treatment while seeking to reduce treatment burden (particularly inappropriate polypharmacy), promoting self-management, sharing decisions with patients, and agreeing an individualised care plan. Services should be delivered by a multi-disciplinary team in the community, but with one clearly defined professional with responsibility for coordinating care. These changes require system re-design facilitated by clinical information systems that provide decision support and allow sharing of information between care providers.

**Research in context**

**Evidence before this study**

A Cochrane review of interventions for improving outcomes in patients with multimorbidity in primary care and community settings was published in April, 2012 (and updated in 2016). The 2012 version, published before this study began, included ten randomised controlled trials, eight of which focused on patients with a broad range of chronic conditions. Nine of the trials were done in North America and might not be generalisable to other health-care systems. The patient inclusion criteria, types of intervention, and outcomes measured were very varied and the findings were inconclusive. The authors concluded that there was a paucity of research and further pragmatic studies based in primary care settings were needed. They highlighted the need to focus on outcomes that are relevant across diseases, such as quality of life, function, and symptom burden. The updated Cochrane review, published in 2016, included eight additional trials but reached similar conclusions. While this trial was in progress, the National Institute for Health and Clinical Excellence (NICE) also reviewed evidence about the management of multimorbidity. They concluded that the evidence base was inadequate and that trials of alternative approaches to organising care for people with multimorbidity were required that examined the effect of these alternative approaches on important clinical outcomes, quality of life, and cost-effectiveness. NICE recommended that care should reflect each patient's individual needs, preferences for treatments, health priorities, and goals.

**Added value of this study**

To our knowledge, this is the largest trial of an intervention to improve management of multimorbidity in primary care. The intervention, based on a patient-centred care model, reflected the international consensus about strategies most likely to improve patient management. The results show that although the intervention was effective at improving experience of patient-centred care, it was not associated with benefits in quality of life or the burden of illness or treatment.

**Implications of all the available evidence**

We have done an updated review to include trials published since the 2016 Cochrane review, combined with the results of this study. The findings showed that, although different studies used a range of strategies to improve care, it is unlikely that current interventions for multimorbidity have a meaningful effect on patients' quality of life. However, several studies (including the 3D study) have shown improvements in patients' experience of individualised patient-centred care which, as highlighted by NICE, is in itself of major importance as one of the triple aims of health care (alongside improving the health of populations and reducing per capita costs).

Despite broad international support for these ideas, there is little evidence about their effectiveness in improving outcomes for patients with multimorbidity. A systematic review^12^ found few randomised trials of interventions, with many remaining uncertainties about their effect on a range of outcomes. Given the importance of multimorbidity for health services there is an urgent need to assess new models of care.

The aim of this study was to implement, at scale, a new approach to managing patients with multimorbidity in primary care and to assess its effectiveness. The 3D approach is based on a patient-centred care model and encapsulates all the strategies recommended in recent international guidelines.^2^, ^3^, ^7^, ^8^, ^9^

## Methods

### Study design and participants

We did a pragmatic cluster-randomised controlled trial in general practices in the UK, after an external pilot of the intervention in three general practices.

General practices providing National Health Service (NHS) primary medical care were recruited from three areas: Bristol and Greater Manchester in England, and Ayrshire in Scotland. Eligible practices had at least two physicians and 4500 registered patients and used the EMIS electronic medical records system. We used EMIS to identify patients with any of the 17 major chronic conditions from those included in the UK Quality and Outcomes Framework (QOF) pay-for-performance programme. We grouped these conditions into ten types of condition with similar management considerations; for example, having two cardiovascular conditions only counted as one condition. Eligible patients were aged 18 years or older, with at least three types of chronic condition. Patients were excluded if they had a life expectancy of less than 12 months, were at serious suicidal risk, were known to be leaving the practice within 12 months, were unable to complete questionnaires in English, were taking part in another health-care research project, lacked the capacity to give consent (in Scotland only, for legal reasons), or if their general practitioner deemed them unsuitable to be invited for other reasons. If more than 150 patients per practice were eligible, a random sample of 150 potential participants were selected, their names were screened by their primary care physicians to exclude patients they judged unsuitable for research, and the remaining patients were invited by post to participate.

All participants (patients and practices) provided written informed consent. The study was approved by South-West England NHS Research Ethics Committee (14/SW/0011) and was done in accordance with the published protocol.^13^

### Randomisation and masking

Patients were assessed for eligibility and invited to participate before practice allocation, and were not informed of their practice's allocation until they had given consent and completed baseline measures. Practices were randomly allocated to provide either the 3D approach or usual care for patients with multimorbidity. Random allocation of practices (clusters) was stratified by area and minimised by practice deprivation and list size. The randomisation system was run from the Bristol Randomised Trials Collaboration by the trial statistician, who was masked to practice identifiers. Allocations were done in blocks of two in each area, with an intervention and a control practice allocated simultaneously so that concealment of allocation was maintained. Patients were informed of their allocation by post, by the research team. Because of the nature of the intervention, practices and participants were aware of their treatment allocation. Outcome data were self-reported or based on automated extraction of data from the electronic medical records, except for details of hospital use, which were collected manually by researchers who were aware of practice allocation. Analysis was done by the trial statistician (DG), who was masked to allocation, except for details of health-care use, for which masking could not be maintained.

### Procedures

General practices in the control group continued to provide usual care. In the UK, review of chronic conditions is mainly done by nurses in primary care, using disease-specific data-entry screens or templates. Nurses often specialise in particular conditions and review each disease separately, so patients with multimorbidity might be invited to multiple review appointments and receive poor continuity of care. Their chronic disease reviews mainly focus on meeting the requirements of the UK Quality and Outcomes Framework pay-for-performance scheme.

**Figure 1:**
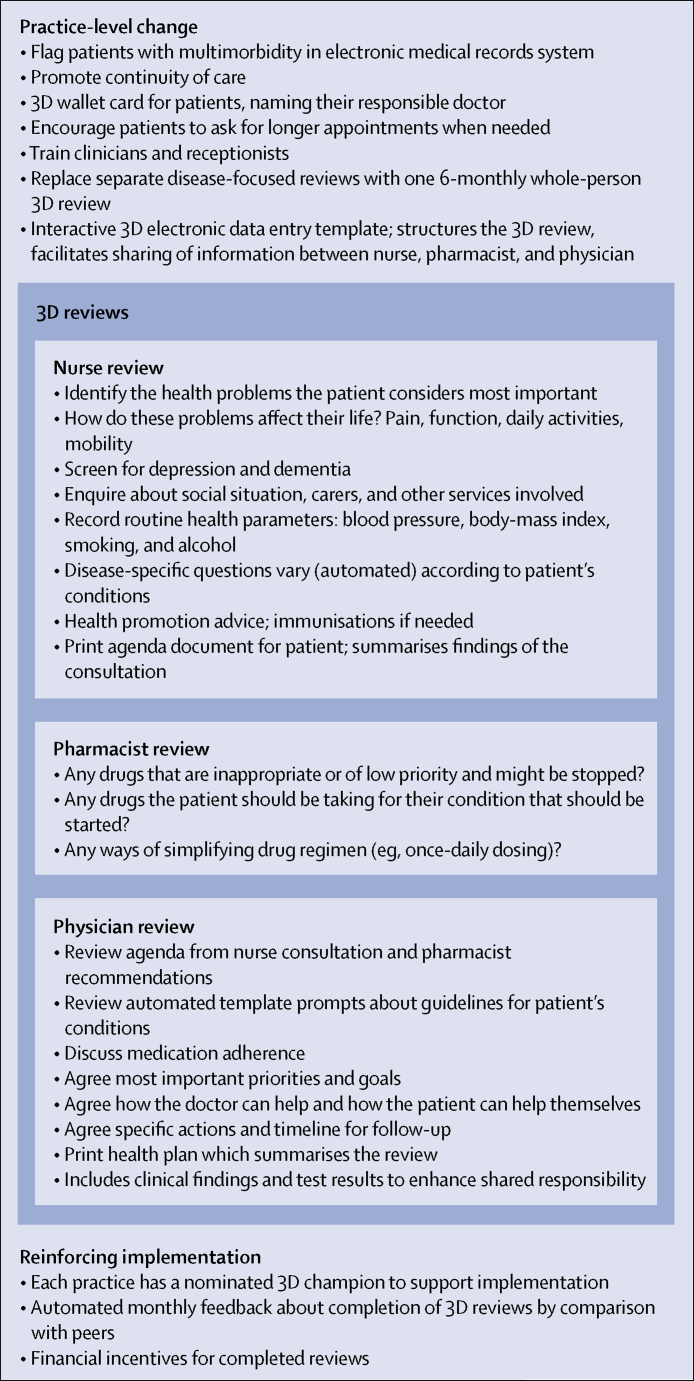
Overview of 3D intervention

The 3D intervention is based on a patient-centred care model and seeks to improve continuity, coordination, and efficiency of care by replacing disease-focused reviews of each health condition with one 6-monthly comprehensive multidisciplinary review (figure 1). The name 3D reminds clinicians to consider dimensions of health in a broad sense, depression, and drugs, while also alluding to the multi-dimensional holistic approach.

Each 3D review consists of two appointments (with a nurse and then a named responsible physician, both existing members of practice staff) and a records-based medication review by a pharmacist (who might or might not have previously worked with the practice). The appointment letter asks the patient to think about the health problems that bother them most. The nurse focuses on identifying the health problems most important to the patient; asking about pain, function, and quality of life; screening for depression and dementia; and then addressing the disease-specific care the patient requires. Findings are printed as a patient-held agenda to inform the subsequent consultation with the doctor. The pharmacist uses the patient's electronic medical records to review medication, and makes recommendations about simplifying and optimising treatment. The physician considers the nurse and pharmacist reviews, discusses treatment adherence, and agrees on a collaborative health plan with the patient. The patient is given a printed copy of the plan, which specifies how the patient and clinicians will address the agreed goals over the next 6 months through routine consultations. All three stages of the 3D review are based on an electronic template integrated within the EMIS electronic medical records system, which reinforces the patient-centred approach and is interactive, with prompts presented to the clinicians that change depending on the patient's combination of chronic conditions.

We used several evidence-based^14^ strategies to facilitate implementation of the 3D approach. All practice clinical staff involved in delivering the intervention received two half-days of training, and administrative staff were trained in a separate meeting. Further details of training are described in the appendix. Each practice identified a local champion to support implementation. We provided practices with monthly feedback about the extent of completion of reviews, and modest financial incentives (£30) for each completed 3D review.

### Outcomes

The primary outcome was health-related quality of life, measured using the EQ-5D-5L instrument.^15^ EQ-5D-5L is a generic instrument comprised of five questions about mobility, self-care, usual activities, pain and discomfort and anxiety and depression, each measured on a five-point scale from no problems to extreme problems.

We chose quality of life as the primary outcome because disease-specific outcomes were not useful in a patient population with different combinations of conditions, and also because the ultimate aim of the 3D intervention was to improve health-related quality of life rather than disease control. We also collected data about secondary outcomes within three domains: illness burden, treatment burden, and patient-centred care. To assess the burden of illness we used a single question about self-rated health, the Bayliss measure of how much illness affects the individual's life,^16^ and the Hospital Anxiety and Depression score^17^ to assess mental health, since multimorbidity is associated with poor mental health.^1^ We designed and validated a new instrument to assess treatment burden^18^ in the absence of a suitable existing measure, assessed medication adherence using the Morisky Medication Adherence eight-item score,^19^ and assessed the number of different drugs prescribed in light of the aim to simplify inappropriate polypharmacy. We assessed patient-centred care using the Patient Assessment of Care for Chronic Conditions (PACIC) measure,^20^ the Consultation and Relational Empathy (CARE) measure of relational empathy,^21^ and single questions (adapted from the NHS Long Term Conditions 6 questionnaire^22^ and the NHS General Practice Patient Survey^23^) about the proportion of patients reporting care related to their priorities, those experiencing their care as joined up, those reporting having a written care plan, and overall satisfaction with care.

We also defined several key care processes as secondary outcomes. We assessed continuity of care using the Continuity of Care index,^24^ which ranges from zero (no continuity) to one (perfect continuity) and the Visit Entropy measure,^25^ in which higher values indicate greater discontinuity. We also recorded numbers of consultations in both primary and secondary care. Additionally, we assessed a summary of disease-specific measures (including measures of disease management and disease control) by measuring the proportion of UK Quality and Outcomes Framework chronic disease targets applicable to each patient that were met.^26^ Additionally, we measured the number of indicators of high-risk prescribing for each patient using an approach developed for a previous trial.^27^ Finally, cost-effectiveness and carer experience were pre-specified outcomes but will be reported separately, as will a parallel qualitative process assessment.

Outcomes were collected at baseline and 9 and 15 months (the primary timepoint) after recruitment. Data were collected by postal questionnaire, with collection of the primary outcome by telephone in non-respondents. Data about patient baseline characteristics, drugs prescribed and process measures were extracted anonymously from patients' routine electronic medical records, except for details of hospital admissions and out-patient attendances, which were collected manually from these medical records.

In light of the nature of the patient population, deaths were anticipated. Patients' primary care physicians reported full details of each death, including the date, cause, and expectedness of each death, any changes to management at the most recent chronic disease review, and any possible association between the intervention and the death. These details were discussed with the independent data monitoring committee.

The protocol originally stated that follow-up timepoints would be at 6 and 12 months. The only change made to the trial protocol was that the follow-up timepoints were changed to 9 and 15 months, before any patients reached the first follow-up timepoint. This change was because we noted a time lag of approximately 3 months between when practices were randomly allocated and when they started to deliver the intervention, because of the need to arrange training and set up the new processes within each practice.

### Statistical analysis

The study was designed with 90% power (with a two-sided α of 0·05) to detect a difference of 0·274 SDs in the EQ-5D-5L. At the time of planning, variability of the new EQ-5D-5L was not available; 0·274 SDs equates to the minimum clinically important difference of 0·074 on the EQ-5D-3L.^28^ Assuming 108 eligible patients per practice, 40% agreeing to participate, 80% retention, and an intra-cluster coefficient of 0·03, we needed to recruit 32 practices and 1382 patients.

We did an intention-to-treat analysis in line with a prespecified analysis plan for all outcomes, using Stata (version 14). All patients were analysed in the groups to which their participating practices were allocated. For analysis of the primary outcome, deceased patients were given an EQ-5D-5L value of zero and missing data were multiply imputed. We used multiple imputation by chain equations including baseline, 9-month, 15-month, and EQ-5D-5L data as available, intervention group, stratification and minimisation variables, and other covariates that were informative of missingness. We converted the EQ-5D-5L scores to an overall index using the van Hout method, with 1 representing full health, zero equivalent to death, and a negative score worse than death.^29^

We analysed all outcomes using multi-level regression models (using linear, logistic, ordinal, or Poisson regression, as appropriate), which included adjustment for baseline measures of the outcome, and stratification and minimisation variables, with practice as a random effect. We also adjusted models considering time-to-death for baseline EQ-5D-5L, age, number of chronic conditions, and length of time in trial. We did prespecified sensitivity analyses of the primary outcome to investigate the influence of excluding missing data, treating EQ-5D-5L as either missing or zero in patients who had died, and adjusting for delays in return of the 15-month follow-up questionnaire. We also considered prespecified subgroups of patient age group, deprivation status, and number of chronic conditions. We analysed secondary outcomes according to the arm to which participants were allocated, without imputation of missing data. Finally, we did a complier average causal effect (CACE) analysis to assess whether greater attendance at 3D review consultations was associated with improvements in the primary outcome. This approach included two analyses with a dichotomous indicator variable for compliance; one analysis amalgamated participants with full attendance (two full 3D reviews with nurse and general practitioner [GP]) or partial attendance (at least one review with nurse or GP); the other combined those with partial or no attendance. The CACE estimates were obtained using instrumental variable regression including the same variables used in the primary analyses, randomised group as an instrumental variable, and the indicator variable for compliance.

Conduct of the trial was monitored by independent trial steering and data monitoring committees. This trial is registered as an International Standard Randomised Controlled Trial, number ISRCTN06180958.

### Role of the funding source

The funder of the study had no role in study design, data collection, data analysis, data interpretation, or writing of the report. The corresponding author had full access to all the data in the study and had final responsibility for the decision to submit for publication.

## Results

**Figure 2:**
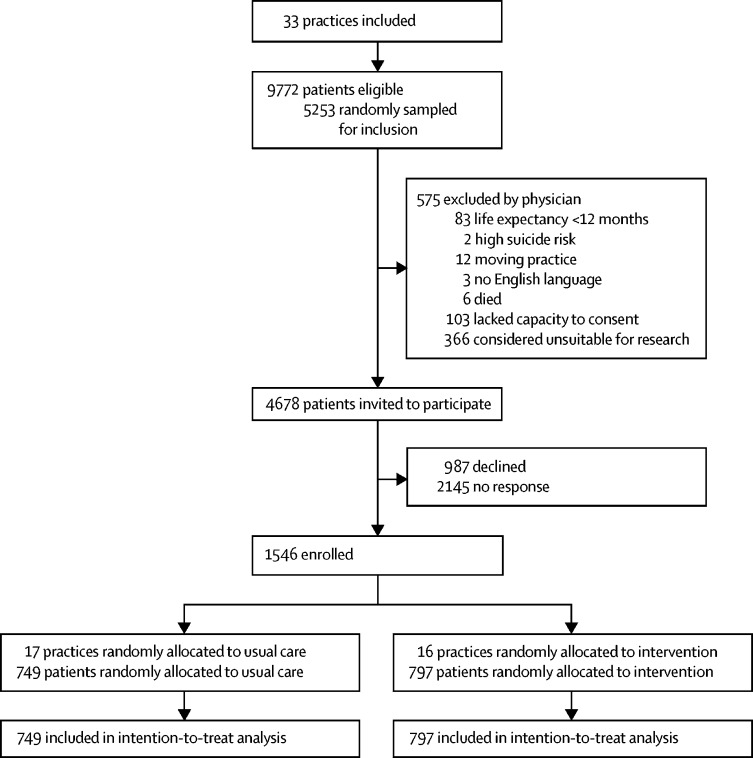
Trial profile

Between Feb 5, 2015, and Sept 21, 2015, we recruited 33 practices which had a total of 248 488 registered adult patients. 9772 (3·9%) were potentially eligible because they were adults aged 18 years or older with three or more types of chronic condition. Of these patients, 5253 were randomly sampled to be screened by their doctors against the exclusion criteria and 4678 eligible participants were invited to participate, as per protocol (figure 2). Between May 20, 2015, and Dec 31, 2015, we recruited 1546 patients, 797 of whom were from 16 practices allocated to the 3D intervention, and 749 patients from 17 practices allocated to usual care.

Participating patients had similar characteristics to non-participants, apart from being less likely to have dementia (appendix p 3). Both practices and patients had similar characteristics at baseline in each study group (table 1; appendix p 4). Patients were predominantly elderly (mean age 71 years) and had a mean of three chronic conditions from those defined in the inclusion criteria, but had a median of seven self-reported conditions from a wider list of 27 chronic conditions included in the Bayliss measure of illness burden^16^ (table 1). 1002 (66%) of 1524 participants rated their health as fair or worse. More than 90% of participants had a cardiovascular condition, about half had diabetes, and half had asthma or chronic obstructive pulmonary disease, while approximately a third of patients had depression (table 1). At baseline, many patients reported deficiencies in their care with respect to whether they had an opportunity to discuss the problems most important to them, whether their care was joined-up, and whether they were satisfied with care. Only 10% of patients reported having a care plan (appendix p 4). We intend to publish elsewhere more details of baseline characteristics of patients and practices, in order to compare participants and non-participants, to establish the external generalisability of our trial, and also to provide more information about usual care for multimorbidity.^30^

**Table 1:** Baseline characteristics

		**Usual care group (n=17 practices, n=749 patients)**	**Intervention group (n=16 practices, n=797 patients)**
**Practices**			
Mean practice list size		9027·2 (4315·6)	9619·2 (3880·2)
Deprivation*			
	Mean IMD for English practices, number of practices	15·8 (12·2), n=12	15·6 (9·6), n=11
	Mean IMD for Scottish practices*, number of practices	26·4 (18·3), n=5	24·2 (20·0), n=5
**Patients**			
Mean age, years		70·7 (11·4)	71·0 (11·6)
Sex			
	Female	377 (50%)	406 (51%)
	Male	372 (50%)	391 (49%)
Ethnicity			
	White	729 (97%)	775 (97%)
	Other or unknown ethnicity	20 (3%)	22 (3%)
Work status			
	Fully retired from work	512 (68%)	525 (66%)
	Other or unknown occupational status	237 (32%)	272 (34%)
Median number of long-term conditions from QOF		3·0 (3·0–3·0)	3·0 (3·0–3·0)
Median number of self-reported conditions, number of patients		7·0 (5·0–10·0), n=748	7·0 (5·0–9·0), n=795
Long-term conditions†			
	Cardiovascular disease or chronic kidney disease‡	698 (93%)	747 (94%)
	Stroke or transient ischaemic attack	241 (32%)	286 (36%)
	Diabetes	401 (54%)	411 (52%)
	Chronic obstructive pulmonary disease or asthma	382 (51%)	388 (49%)
	Epilepsy	35 (5%)	41 (5%)
	Atrial fibrillation	249 (33%)	281 (35%)
	Serious mental illness§	37 (5%)	29 (4%)
	Depression	283 (38%)	276 (35%)
	Dementia	27 (4%)	33 (4%)
	Learning disability	7 (1%)	7 (1%)
	Rheumatoid arthritis	55 (7%)	48 (6%)
Mean EQ-5D-5L score, number of patients		0·542 (0·292), n=747	0·574 (0·282), n=795

Data are mean (SD), n (%), n/N (%), or median (IQR). IMD=Index of Multiple Deprivation. QOF=UK Quality and Outcomes Framework.

* English and Scottish IMD scores are based on similar variables but calculated differently; data available as mean score for English practices, but proportion of patients living in 15% most deprived data zones for Scottish practices.

† Conditions with similar clinical management were grouped and only counted once.

‡ Including coronary heart disease, hypertension, heart failure, peripheral arterial disease, and chronic kidney disease stages 3 to 5.

§ Including schizophrenia, psychosis, and bipolar disease.

Primary outcome data were available for 1361 (88%) participants at 15 months follow-up (n=670 in the usual care group and n=691 in the intervention group). There was no evidence of a difference between study groups in health-related quality of life in the primary analysis, with missing data imputed (adjusted difference in means 0·00 (95% CI −0·02 to 0·02); p=0·93; table 2).

**Table 2:** Outcomes at 15 months

		**Usual care group (N=749)**	**Intervention group (N=797)**	**Adjusted difference in means (95% CI); p value**
Primary outcome*				
Unadjusted mean EQ-5D-5L (SE)		0·504 (0·012)	0·533 (0·012)	0·00 (−0·02 to 0·02); p=0·93†
**Secondary outcomes**				
Illness burden				
	Self-rated health of good or better‡	230/631 (36%)	242/642 (38%)	0·84§ (0·67 to 1·05); p=0·13
	Mean Bayliss measure of illness burden^16^	18·4 (12·9); n=590	16·7 (11·6); n=598	−0·64¶ (−1·54 to 0·27); p=0·17
	Mean HADS anxiety score^17^	6·3 (4·8); n=624	5·8 (4·7); n=629	−0·24¶ (−0·57 to 0·08); p=0·15
	Mean HADS depression score^17^	6·8 (4·6); n=625	6·1 (4·6); n=630	−0·01¶ (−0·33 to 0·30); p=0·94
Treatment burden				
	Mean Multimorbidity Treatment Burden Questionnaire score^18^	15·0 (17·1); n=626	12·9 (15·0); n=625	−0·46¶ (−1·78 to 0·86); p=0·49
	Mean eight-item Morisky Medication Adherence Score^19^	6·6 (1·3); n=749	6·7 (1·2); n=797	0·06¶ (−0·05 to 0·17); p=0·27
	Median number of different drugs prescribed in past 3 months	11·0 (8·0–15·0); n=736	11·0 (8·0–15·0); n=774	1·02‖ (0·97 to 1·06); p=0·46
Patient-centred care				
	Mean PACIC score^20^	2·5 (0·9); n=512	2·8 (1·0); n=524	0·29¶ (0·16 to 0·41); p<0·0001
	Mean CARE doctor score^21^	37·5 (10·0); n=601	40·2 (9·7); n=617	1·20¶ (0·28 to 2·13); p=0·0109
	Mean CARE nurse score^21^	38·5 (9·5); n=462	40·8 (8·9); n=535	1·11¶ (0·03 to 2·19); p=0·044
	Patients reporting they almost always discuss the problems most important to them in managing their own health‡	153/599 (26%)	256/612 (42%)	1·85§ (1·44 to 2·38); p<0·0001
	Patients reporting that support and care is almost always joined-up‡	173/603 (29%)	257/614 (42%)	1·48§ (1·18 to 1·85); p=0·0006
	Patients reporting being very satisfied with care‡	236/608 (39%)	345/614 (56%)	1·57§ (1·19 to 2·08); p=0·0014
	Patients reporting having a written care plan, health plan, or treatment plan**	91/623 (15%)	141/623 (23%)	1·97†† (1·32 to 2·95); p=0·0010

Data are n/N (%), median (IQR), or mean (SD), unless otherwise indicated; treatment effects are presented as adjusted odds ratios, beta-coefficients, or incidence rate ratios (see footnotes). Use of the Morisky Medication Adherence Score is protected by US Copyright laws. Permission for use is required. A licence agreement is available from Donald E Morisky, MMAS Research LLC, 14725 NE 20th St, Bellevue, WA 98007, USA, or from dmorisky@gmail.com. HADS=Hospital And Depression Score.

* Means calculated with multiple imputation by chain equations.

† Intracluster correlation coefficient was 0·00 (95% CI 0·00–0·00).

‡ Ordinal variable, dichotomised for ease of presentation; full details of question and responses available in appendix.

§ Adjusted odds ratio from multi-level ordinal logistic regression.

¶ Beta-coefficients; analyses are adjusted multi-level linear regression models.

‖ Incidence rate ratio from adjusted multi-level Poisson regression model.

** Not prespecified in the statistical analysis plan; responses of “don't know” were treated as not having a care plan.

†† Adjusted odds ratio from multi-level logistic regression.

Results for measures of illness burden, treatment burden, and patient-centred care at 15 months follow-up are shown in table 2 (see appendix pp 7–8 for 9-month data and full details of ordinal outcomes). There was no evidence of difference between intervention and usual care groups with respect to measures of illness burden, including self-rated health, anxiety, or depression, or the effect of illness on life. There was also no evidence that the intervention reduced patient-reported treatment burden, the number of drugs prescribed, or improved medication adherence.

All measures of patient-centred care showed benefits from the intervention after 15 months, namely the PACIC measure,^20^ the CARE measure,^21^ the proportion of patients reporting care related to their priorities, those reporting care as joined-up, those reporting a written care plan, and overall satisfaction with care (table 2).

Results for process of care measures are shown in table 3. There was a significant difference in the Continuity of Care Index^24^ in the intervention group compared with the usual care group (table 3).^25^ There was no evidence of a difference between intervention and usual care in quality of disease management (ie, number of QOF indicators met)^26^ or the number of indicators of high-risk prescribing.^27^

**Table 3:** Process of care outcomes at 15 months

	**Usual care group (N=749)**	**Intervention group (N=797)**	**Adjusted difference (95% CI); p value**
Mean Continuity of Care index*	0·3 (0·3); n=720	0·4 (0·3); n=769	0·08† (0·02 to 0·13); p=0·0045
Mean Visit Entropy‡	107·3 (79·3); n=720	99·3 (72·7); n=769	−8·76† (−18·07 to 0·55); p=0·07
Mean number of QOF indicators met (quality of disease management)	85·6 (17·3); n=475	84·3 (17·5); n=493	0·41† (−3·05 to 3·87); p=0·82
Median number of indicators of high-risk prescribing	0·0 (0·0, 1·0); n=741	0·0 (0·0, 1·0); n=780	1·04§ (0·87 to 1·25); p=0·68
Median number of primary care physician consultations	8·0 (4·0, 14·0); n=739	10·0 (6·0, 16·0); n=778	1·13§ (1·02 to 1·25); p=0·0209
Median number of nurse consultations	4·0 (2·0, 8·0); n=739	6·0 (4·0, 10·0); n=778	1·37§ (1·17 to 1·61); p=0·0001
Median number of hospital admissions	0·0 (0·0, 1·0); n=743	0·0 (0·0, 1·0); n=785	1·04§ (0·84 to 1·30); p=–0·71
Median number of hospital outpatient attendances	2·0 (1·0, 5·0); n=743	3·0 (1·0, 5·0); n=785	1·02§ (0·92 to 1·14); p=0·72

Data are mean (SD) or median (IQR), unless otherwise indicated. QOF=UK Quality and Outcomes Framework.

* Ranges from 0 to 1, with 0 indicating no continuity of care (patient saw a different provider at each consultation) and 1 indicating perfect continuity of care (patient saw the same provider at each consultation).^24^

† Beta-coefficients; analyses are adjusted multi-level linear regression models.

‡ Range from 0 to –log_2_(1/κ), where κ is the total number of care providers visited, with the minimum of 0 indicating perfect continuity of care and the maximum of –log_2_(1/κ) indicating no continuity of care.^25^

§ Incident rate ratio from adjusted multi-level Poisson model; exposure covariate is per patient length of time in trial.

Patients in the intervention group had more nurse consultations and more primary care physician consultations over 15 months, compared with the usual care group (table 3). There was no evidence of difference in the number of hospital admissions or outpatient attendances (table 3).

595 (75%) of 797 patients received at least one 3D review over 15 months, and 390 (49%) had two complete 3D reviews as intended. When patients attended the reviews, these were well completed by clinicians (appendix). Individual elements of the reviews were completed in the vast majority of consultations for which they were relevant, except for printing the 3D health plan, which followed 461 (77%) of 598 GP 3D review consultations (appendix p 9). Furthermore, 607 (76%) of all patients in the intervention group had a review of their medication by the pharmacist. By comparison, patients in the usual care group attended disease-specific reviews for their chronic conditions on 702 (78%) of 897 occasions when a review was required by the UK Quality and Outcomes Framework. There was no evidence from the CACE analysis that patients who attended two 3D reviews were more likely to have an improvement in quality of life (appendix p 10).

During the trial, 78 (5%) of 1546 patients died, including 46 (6%) of 797 patients in the intervention group and 32 (4%) of 749 in the usual care group. There was no evidence of difference in the number of deaths between the intervention and usual care groups (χ^2^ 1·811; p=0·18; Cox proportional hazards ratio 1·39, 95% CI 0·92–2·08; p=0·11). None of the deaths were reported by the patient's general practitioner as possibly related to the intervention, and investigation of the causes of death and recent changes in patient management did not suggest any relationship with the intervention.

The results for the primary outcome were consistent in all sensitivity analyses (appendix p 5) and we found no evidence of differential effect in any of our predefined subgroup analyses at 15 months (appendix p 6).

## Discussion

We investigated the 3D approach, which was based on a patient-centred care model and which implements strategies recommended in international guidelines for the management of patients with multimorbidity.^2^, ^3^, ^7^, ^8^, ^9^ Although the intervention was not associated with an improvement in quality of life or the secondary outcomes of perceived illness burden or treatment burden, and had mixed effects on the process of care, it was associated with significant improvements in measures of patient-centred care.

This trial has several strengths. It is the largest trial of an intervention to improve management of multimorbidity, and was rigorously done in line with recommended standards for cluster-randomised trials. We recruited a population of patients with major health needs: two-thirds of participants rated their health as fair or worse and many reported clear deficiencies in the care of their chronic conditions. The use of broad eligibility criteria in a range of practices in different settings enhances external validity. The findings are likely to be generalisable to other countries where patients have chronic disease reviews focused on management of individual diseases. The trial was highly pragmatic and reflects the effectiveness of the intervention in real-world implementation.^31^

The pragmatic nature of the trial reflects the delivery of care in routine settings, and only 49% of intervention participants received two 3D reviews as intended, which dilutes the potential effectiveness of the approach. However, three-quarters of patients in the intervention group had at least one review, which is similar to the extent to which patients received chronic disease reviews under usual care, indicating that the 3D model was implemented as well as were current models of care. Second, only a third of invited patients agreed to participate. This recruitment rate is typical of trials in this population,^32^ but raises the possibility of recruitment bias. It is reassuring that participating patients had similar characteristics to non-participants, apart from being less likely to have dementia. Third, potential participants were selected on the basis of them having three or more major long term conditions, rather than having poor quality of life. This approach would reduce the capacity of the trial to show benefit in patients who had few problems at baseline (although as previously noted most participants had poor health). Fourth, there was some chance imbalance in the primary outcome at baseline, but this was adjusted for in the analyses. Finally, the large number of secondary outcomes raises the possibility of false-positive findings due to multiple testing, and the fact that most outcomes were based on patient self-report raises potential for bias in this unblinded trial. However, in pragmatic trials it is important to collect data for a broad range of outcomes of relevance to patients, clinicians, and policy makers,^31^ and the consistent results in every outcome domain adds confidence to our interpretation of the findings. An initiative to develop a core outcome set for trials in multimorbidity highlighted the wide range of outcomes that need to be considered, and this trial includes most of these outcomes.^33^

Assessing disease-specific outcomes in multimorbidity is problematic because participants have different combinations of conditions, but we addressed this by assessing the proportion of disease-specific indicators from the UK Quality and Outcomes Framework relevant to each patient that were met.^26^ We were interested in the possibility that a greater focus on patient's individual priorities and shared decision making might lead to worse disease-specific outcomes, but there was no evidence for this.

We recognise that the 3D intervention might have supported changes in organisation more than it supported changing the clinicians' attitudes on which patient-centredness depends. However, the changes introduced by the 3D intervention were sufficient for participants to report improvements in patient-centred care. For example, the PACIC questionnaire measures patients' experience of specific actions, such as being given a written list of things they could do to improve their health, which reflect high-quality care for chronic disease.^20^ It is possible that the 3D intervention was not sufficiently intensive to affect entrenched problems such as difficulties with mobility or self-care, which are measured by the EQ-5D-5L questionnaire. However, other related but much more intensive interventions, such as Guided Care, have also not shown evidence of improved quality of life.^32^ Possible explanations for these findings could be that current quality-of-life measurement tools are not sufficiently sensitive to detect meaningful changes or that multimorbidity interventions need to be provided over a longer period than studied in this trial before benefits become apparent.

An alternative interpretation of our findings is that the causal model underlying the international consensus is flawed. This interpretation assumes that better patient-centred health care for patients with multimorbidity will result in better health and wellbeing. The updated Cochrane systematic review^12^ of trials of interventions for multimorbidity showed heterogeneity in terms of the interventions, inclusion criteria, outcomes, and effects. Results were mixed and inconclusive, with little evidence that interventions for multimorbidity improved clinical outcomes or quality of life, apart from modest improvements in mental health outcomes in studies that targeted patients with depression alongside physical health problems.

We have updated the findings of this 2016 Cochrane review to include more recent studies that reported quality of life as an outcome, and identified eight additional studies, including this study (appendix p 11). Meta-analysis of the results (which should be interpreted with caution in view of heterogeneity in type of intervention and patient inclusion criteria) provides stronger evidence that interventions for multimorbidity are associated with little or no meaningful benefit in quality of life. Furthermore, a funnel plot suggests the possibility of publication bias, with all of the largest trials showing no evidence of benefit whereas several small studies, some of which are pilot studies, provide positive findings (appendix p 12).

It is possible that the 3D intervention, just as with other multimorbidity interventions such as Guided Care,^32^ improves patients' perceptions of the quality of their care but not the quality of their lives. Improving patient experience is one of the triple aims of health care,^34^ so providing care that is demonstrably more patient-centred is arguably sufficient justification for implementation in itself, especially since our evidence shows it is not associated with disadvantages in terms of disease management or hospital use. This argument is based on the premise that health care should respect the priorities and preferences of patients, and this should be reflected in the tools used to assess health-care quality.^35^

## References

[1] Barnett K, Mercer SW, Norbury M, Watt G, Wyke S, Guthrie B (2012). Epidemiology of multimorbidity and implications for health care, research, and medical education: a cross-sectional study. Lancet.

[2] US Department of Health and Human Services (2010). Multiple chronic conditions—a strategic framework: optimum health and quality of life for individuals with multiple chronic conditions.

[3] National Guideline Centre (2016). Multimorbidity: clinical assessment and management.

[4] Salisbury C (2012). Multimorbidity: redesigning health care for people who use it. Lancet.

[5] May C, Montori VM, Mair FS (2009). We need minimally disruptive medicine. BMJ.

[6] Bayliss EA, Edwards AE, Steiner JF, Main DS (2008). Processes of care desired by elderly patients with multimorbidities. Fam Pract.

[7] Boyd CM, McNabney MK, Brandt N (2012). Guiding principles for the care of older adults with multimorbidity: an approach for clinicians: American Geriatrics Society expert panel on the care of older adults with multimorbidity. J Am Geriatr Soc.

[8] Palmer K, Marengoni A, Forjaz MJ (2018). Multimorbidity care model: rjecommendations from the consensus meeting of the Joint Action on Chronic Diseases and Promoting Healthy Ageing across the Life Cycle (JA-CHRODIS). Health Policy.

[9] Muth C, van den Akker M, Blom JW (2014). The Ariadne principles: how to handle multimorbidity in primary care consultations. BMC Med.

[10] Stewart M, Brown JB, Weston WW, McWhinney IR, McWilliam CL, Freeman TR (2014). Patient-centred medicine: transforming the clinical method.

[11] Bodenheimer T, Wagner EH, Grumbach K (2002). Improving primary care for patients with chronic illness. JAMA.

[12] Smith SM, Wallace E, O'Dowd T, Fortin M (2016). Interventions for improving outcomes in patients with multimorbidity in primary care and community settings. Cochrane Database Syst Rev.

[13] Man MS, Chaplin K, Mann C (2016). Improving the management of multimorbidity in general practice: protocol of a cluster randomised controlled trial (the 3D Study). BMJ Open.

[14] Lau R, Stevenson F, Ong BN (2015). Achieving change in primary care—effectiveness of strategies for improving implementation of complex interventions: systematic review of reviews. BMJ Open.

[15] Herdman M, Gudex C, Lloyd A (2011). Development and preliminary testing of the new five-level version of EQ-5D (EQ-5D-5L). Qual Life Res.

[16] Bayliss EA, Ellis JL, Steiner JF (2009). Seniors' self-reported multimorbidity captured biopsychosocial factors not incorporated into two other data-based morbidity measures. J Clin Epidemiol.

[17] Zigmond AS, Snaith RP (1983). The hospital anxiety and depression scale. Acta Psychiatr Scand.

[18] Duncan P, Murphy M, Man MS, Chaplin K, Gaunt D, Salisbury C (2018). Development and validation of the Multimorbidity Treatment Burden Questionnaire (MTBQ). BMJ Open.

[19] Morisky DE, Ang A, Krousel-Wood M, Ward P (2008). Predictive validity of a medication adherence measure in an outpatient setting. J Clin Hypertens.

[20] Glasgow RE, Wagner EH, Schaefer J, Mahoney LD, Reid RJ, Greene SM (2005). Development and validation of the Patient Assessment of Chronic Illness Care (PACIC). Med Care.

[21] Mercer SW, Maxwell M, Heaney D, Watt GC (2004). The consultation and relational empathy (CARE) measure: development and preliminary validation and reliability of an empathy-based consultation process measure. Fam Pract.

[22] The Health Foundation (2016). LTC6 Questionnaire. http://personcentredcare.health.org.uk/resources/ltc6-questionnaire.

[23] Ipsos MORI Social Research Institute (2015). GP patient survey—technical annex. 2014–2015 annual report.

[24] Bice TW, Boxerman SB (1977). A quantitative measure of continuity of care. Med Care.

[25] Garrison GM, Keuseman R, Bania B, Robelia P, Pecina J (2017). Visit entropy associated with hospital readmission rates. J Am Board Fam Med.

[26] Reeves D, Campbell SM, Adams J, Shekelle PG, Kontopantelis E, Roland MO (2007). Combining multiple indicators of clinical quality—An evaluation of different analytic approaches. Med Care.

[27] Dreischulte T, Donnan P, Grant A, Hapca A, McCowan C, Guthrie B (2016). Safer prescribing—a trial of education, informatics, and financial incentives. N Engl J Med.

[28] Walters SJ, Brazier JE (2005). Comparison of the minimally important difference for two health state utility measures: EQ-5D and SF-6D. Qual Life Res.

[29] van Hout B, Janssen MF, Feng Y-S (2012). Interim scoring for the EQ-5D-5L: mapping the EQ-5D-5L to EQ-5D-3L value sets. Value in Health.

[30] Chaplin K, Bower P, Man M-S, et al. Understanding usual care for patients with multimorbidity: baseline data from a cluster randomised trial of the 3D intervention in primary care. *BMJ Open* (in press). 10.1136/bmjopen-2017-019845 PMC6119425 30158215

[31] Ware JH, Hamel MB (2011). Pragmatic trials—guides to better patient care?. N Engl J Med.

[32] Boult C, Leff B, Boyd CM (2013). A matched-pair cluster-randomized trial of guided care for high-risk older patients. J Gen Intern Med.

[33] Smith SM, Wallace E, Salisbury C, Sasseville M, Bayliss E, Fortin M (2018). A core outcome set for multimorbidity research (COSmm). Ann Fam Med.

[34] Berwick DM, Nolan TW, Whittington J (2008). The triple aim: care, health, and cost. Health Aff (Millwood).

[35] Nolte E (2017). Implementing person centred approaches. BMJ.

