# Supplementary material for: Management of multimorbidity using a patient-centred care model: a pragmatic cluster-randomised trial of the 3D approach
Source: Lancet. 2018 Jul 7;392(10141):41–50. doi: 10.1016/S0140-6736(18)31308-4 (PMC6041724; doi:10.1016/S0140-6736(18)31308-4)
Supplement: Supplementary appendix [file mmc1.pdf]

THE LANCET

Supplementary appendix

This appendix formed part of the original submission and has been peer reviewed. We post it as supplied by the authors.

Supplement to: Salisbury C, Man M-S, Bower P, et al. Management of multimorbidity using a patient-centred care model: a pragmatic cluster-randomised trial of the 3D approach. *Lancet* 2018; published online June 28. http://dx.doi.org/10.1016/S0140-6736(18)31308-4.

Web appendix: Improving the management of multimorbidity using a patient-centred care model: pragmatic cluster randomised trial of the 3D approach

Contents

Appendix: Training 2
Appendix Table 1 Comparison between patients invited and recruited 3
Appendix Table 2 Secondary outcome measures at baseline 4
Appendix Table 3 Sensitivity analyses of primary outcome at 15 months follow-up 5
Appendix Table 4 Sub-group analyses of primary outcome at 15 months follow-up 6
Appendix Table 5 Secondary outcome measures at 9 months follow-up 7
Appendix Table 6 Secondary outcomes at 9 and 15 months: full details of ordinal outcomes 8
Appendix Table 7 Completion of the 3D reviews 9
Appendix Table 8· Complier averaged causal effect (CACE) analysis 10
Appendix Updated systematic review incorporating previous trials and this trial 11
Appendix Figure 1 Forest plot of trials of interventions in primary care for patients with multimorbidity 11
Appendix Figure 2 Funnel plot 12
References 13

Appendix: Training

Each member of clinical staff delivering the intervention attended two half days of training. These covered key concepts for the 3D approach. The training included case based discussion, demonstration of the interactive electronic template, reflection, evaluation and 'homework'.

The content included:

- Overview of the 3D approach
- What is patient centred care?
- Why is continuity of care important and how can it be improved?
- The pros and cons of co-ordinated reviews for all health conditions at once
- Identifying the patient's main priorities and concerns
- Screening for depression and the importance of mental health in multimorbidity
- Polypharmacy and medication adherence
- Goal setting and health care planning
- Use of the interactive 3D template

In addition a separate training meeting was held with the practice reception and administrative staff. This covered:

- The importance of continuity of care and strategies for how to support this
- Offering longer appointments when appropriate
- The need to change recall systems, cancelling disease focused reviews for trial patients and inviting them to 3D reviews instead
- How to run and export regular searches about progress with undertaking the 3D reviews

Appendix Table 1 Comparison between patients invited and recruited

	**Patients invited but not randomized (n= 3132)**	**Invited and randomized participants (n=1546)**
Female: n (%)	1680 (54%)	783 (51%)
Age: mean (SD)	71·3 (13·5)	70·8 (11·5)
Total no. of long term conditions: mean (SD)	3·3 (0·5)	3·2 (0·5)
Long term condition: n (%)		
Cardiovascular Disease	2875 (92%)	1445 (93%)
Stroke or TIA	1050 (34%)	527 (34%)
Diabetes	1613 (52%)	812 (53%)
COPD or Asthma	1456 (46%)	770 (50%)
Epilepsy	185 (6%)	76 (5%)
Atrial Fibrillation	928 (30%)	530 (34%)
Mental Health	200 (6%)	66 (4%)
Depression	1250 (40%)	559 (36%)
Dementia	340 (11%)	60 (4%)
Learning Disability	84 (3%)	14 (1%)
Rheumatoid Arthritis	196 (6%)	103 (7%)

Appendix Table 2 Secondary outcome measures at baseline

	Usual care (n=749)	Intervention (n=797)
**Health and illness data**		
Mean EQ-5D-5L score (SD), n	0·542 (0·292), 747	0·574 (0·282), 795
Self-rated health: no. of patients 'good' or above / total no.(%)	231/741 (31%)	291/783 (37%)
Bayliss illness burden score:[1] mean (SD), n	19·5 (12·7), 700	18·2 (12·0), 758
Self-reported chronic conditions: median(IQR), n	7·0 (5·0, 10·0), 748	7·0 (5·0, 9·0), 795
Hospital And Depression Score (HADS) Anxiety score:[2] mean (SD), n	6·4 (4·8), 740	6·1 (4·6), 785
HADS Depression score:[2] mean (SD), n	7·0 (4·5), 743	6·3 (4·2), 791
**Treatment burden**		
Multimorbidity Treatment Burden Questionnaire score: mean (SD), n	15·7 (15·9), 736	13·3 (14·7), 789
Morisky Medication Adherence Score 8 item:[3] mean (SD), n[a]	6·7 (1·4), 749	6·8 (1·4), 797
No. of different drugs prescribed in the three months before baseline: mean (SD), n	11·3 (5·4), 738	11·1 (5·2), 778
**Patient-centred care**		
PACIC score:[4] mean (SD), n	2·4 (1·0), 608	2·6 (0·9), 624
CARE doctor score:[5] mean (SD), n	38·8 (9·8), 714	40·8 (9·1), 781
CARE nurse score:[5] mean (SD), n	39·0 (9·1), 565	40·7 (9·2), 610
Patient discussed most important problems	n=716	n=763
Not at all	145 (20%)	114 (15%)
Rarely	128 (18%)	123 (16%)
Some of the time	249 (35%)	271 (36%)
Almost always	194 (27%)	255 (33%)
Care joined up	n=716	n=763
Not at all	111 (16%)	63 (8%)
Rarely	96 (13%)	69 (9%)
Some of the time	280 (39%)	310 (41%)
Almost always	229 (32%)	321 (42%)
Overall satisfaction	n=722	n=772
Very dissatisfied	20 (3%)	16 (2%)
Fairly dissatisfied	37 (5%)	24 (3%)
Neither satisfied nor dissatisfied	94 (13%)	55 (7%)
Fairly satisfied	251 (35%)	238 (31%)
Very satisfied	320 (44%)	439 (57%)
No. of patients self-reporting having a written care, health or treatment plan: no. of patients/total no. (%)	74/739 (10%)	77/787 (10%)
**Process measures**		
Continuity of care	0·3 (0·3), 712	0·4 (0·3), 767
Continuity of Care index:[6] Mean (SD), n[b,c]		
Visit Entropy:[7] Mean (SD), n[b,d]	101·1 (66·1), 712	103·9 (67·1), 767
Quality of disease management. QOF indicators met:[8] Mean (SD), n[e]	84·5 (18·6), 526	77·2 (23·2), 552
No. of primary care consultations with doctor:[b] median (IQR), n	7·0 (4·0, 11·0), 739	8·0 (5·0, 12·0), 778
No. of primary care consultations with nurse:[b] median (IQR), n	4·0 (2·0, 7·0), 739	4·0 (2·0, 8·0), 778

[a]Use of the ©MMAS is protected by US Copyright laws. Permission for use is required. A licence agreement is available from Donald E Morisky, MMAS Research LLC 14725 NE 20th St Bellevue WA 98007 or from dmorisky@gmail.com

[b]Face to face (home or surgery or nursing home) or phone consultations over the 12-months before recruitment.

[c]Range from 0 to 1 with 0 indicating no continuity of care; patient saw a different provider at each consultation and 1 indicating perfect continuity of care; patient saw the same provider at each consultation.

[d]Range from 0 to $-\log_2(1/k)$, where k is the total number of care providers visited, with the minimum of 0 indicating perfect continuity of care; patient saw the same provider at each consultation and the maximum of $-\log_2(1/k)$ indicating no continuity of care; patient saw a different provider at each consultation.

[e]The % of indicators that were relevant to each patient that were met, averaged across all patients. This is the 'patient average' approach of Reeves et al[8]

Appendix Table 3 Sensitivity analyses of primary outcome at 15 months follow-up

	Usual care		Intervention				
	**EQ-5D-5L unadjusted mean (SD or SE)**	**N**	**EQ-5D-5L unadjusted mean (SD or SE)**	**N**	**Adjusted difference in means[a]**	**95% Confidence Interval**	**P**
**Primary analysis[b]**	0·504 (0·012[c])	749	0·533 (0·012[c])	797	0·00	-0·02, 0·02	0·93
**Sensitivity analysis in relation to missing EQ-5D-5L**							
No multiple imputation; death set as zero	0·517 (0·311)	670	0·546 (0·303)	691	0·00	-0·02, 0·02	0·82
No multiple imputation; death left as missing	0·542 (0·296)	638	0·585 (0·275)	645	0·01	-0·01, 0·02	0·53
Imputation using last observation carried forward, including deceased patients	0·512 (0·310)	749	0·548 (0·300)	797	0·01	-0·01, 0·03	0·37
No multiple imputation; death set as zero; adjusted by days between recruitment and return of 15-month questionnaire	0·517 (0·311)	670	0·546 (0·303)	691	0·01	-0·02, 0·03	0·52

[a] All analyses are multi-level linear regression models adjusted by centre, baseline EQ-5D-5L score, practice list size and deprivation score. Practice is included as a random effect.
[b] Using multiple imputation by chain equations including baseline, 9 month, 15 month and EQ-5D-5L data as available, intervention arm, stratifying/minimisation variables and other covariates that were informative of missingness.
[c] Standard error

Appendix Table 4 Sub-group analyses of primary outcome at 15 months follow-up

	Usual care		Intervention					
	**EQ-5D-5L unadjusted mean (SD)**	**N[a]**	**EQ-5D-5L unadjusted mean (SD)**	**N[a]**	**Adjusted difference in means[b]**	**95% Confidence Interval**	**Interaction term (95% CI)**	**Interaction term P-value**
**Participants by median age**								
<72 years	0·532 (0·321)	334	0·566 (0·312)	324	0·00	-0·03, 0·03		
≥72 years	0·501 (0·301)	336	0·529 (0·295)	367	0·00	-0·03, 0·03	0.00 (-0.04, 0.05)	0·87
**Number of long-term conditions**								
Three	0·539 (0·305)	534	0·581 (0·285)	558	0·01	-0·02, 0·03		
Four or more	0·428 (0·320)	136	0·402 (0·334)	133	-0·05	-0·09, 0·00	-0.05 (-0.11 ,0.00)	0·05
**Deprivation**								
England: Quartiles of IMD score[c]								
1st quartile	0·569 (0·290)	119	0·633 (0·277)	124	0·04	-0·01, 0·10		
2nd quartile	0·537 (0·345)	140	0·568 (0·298)	115	-0·04	-0·09, 0·01	-0.08 (-0.15, -0.01)	
3rd quartile	0·563 (0·285)	105	0·536 (0·305)	127	-0·03	-0·08, 0·03	-0.07 (-0.15, 0.00)	
4th quartile	0·465 (0·310)	118	0·497 (0·296)	122	0·01	-0·05, 0·06	-0.04 (-0.11, 0.04)	0·11[d]
Scotland: Quartiles of SIMD score[c]								
1st quartile	0·506 (0·306)	47	0·583 (0·316)	55	0·06	-0·02, 0·14		
2nd quartile	0·499 (0·312)	34	0·502 (0·324)	56	-0·08	-0·16, 0·01	-0.13 (-0.25, -0.02)	
3rd quartile	0·456 (0·321)	48	0·494 (0·311)	52	-0·01	-0·09, 0·07	-0.07 (-0.18, 0.05)	
4th quartile	0·450 (0·291)	59	0·476 (0·311)	40	0·02	-0·07, 0·10	-0.04 (-0.15, 0.07)	0·16[d]
**Depression**								
No	0·536 (0·318)	424	0·571 (0·289)	458	0·00	-0·02, 0·03		
Yes	0·483 (0·297)	246	0·498 (0·326)	233	-0·02	-0·05, 0·02	-0.02 (-0.07, 0.03)	0·40

[a] Numerator includes those who died, who were attributed an EQ-5D-5L score of zero
[b]All analyses are multi-level linear regression models adjusted by centre, baseline EQ-5D-5L score, practice list size and deprivation score. Practice is included as a random effect.
[c]Using participant postcode matched to England IMD data 2010 or Scotland SIMD data from 2012
[d]p-value from likelihood ratio test comparing model with interaction term against model without interaction term

Appendix Table 5 Secondary outcome measures at 9 months follow-up

	Usual care	Intervention	Adjusted difference	95% CI	P-value
**Health and illness data**					
EQ-5D-5L: mean (SD), n	0·526 (0·306), 684	0·566 (0·294), 699	0·01	-0·01, 0·03	0·53
Self-rated health: no. of patients rating 'good' or above/ total no (%)[a]	237/666 (36%)	268/672 (40%)	0·95[b]	0·76, 1·19	0·66
Bayliss measure of illness burden:[1] mean (SD), n	18·1 (12·8), 611	17·6 (13·0), 636	0·30[c]	-0·65, 1·26	0·54
Hospital And Depression Score (HADS) Anxiety score:[2] mean (SD), n	6·1 (4·7), 638	5·7 (4·6), 652	-0·18[c]	-0·50, 0·14	0·26
HADS Depression score: mean (SD), n	6·6 (4·5), 641	6·1 (4·4), 654	0·07[c]	-0·22, 0·36	0·65
**Treatment burden**					
Multimorbidity Treatment Burden Questionnaire score: mean (SD), n	14·4 (16·0), 640	12·1 (14·8), 658	-1·09[c]	-2·29, 0·12	0·08
Morisky Medication Adherence Score 8 item:[3] mean (SD), n [d]	6·6 (1·4), 749	6·7 (1·3), 797	-0·03[c]	-0·14, 0·08	0·55
**Patient-centred care**					
PACIC score:[4] mean (SD), n	2·4 (0·9), 554	2·7 (1·0), 556	0·28[c]	0·18, 0·38	<0·0001
CARE doctor score:[5] mean (SD), n	37·5 (10·2), 632	40·6 (9·8), 649	1·44[c]	0·47, 2·41	0.0035
Patient discussed most important problems: No. reporting "almost always"/ total no. (%)[a]	167/634 (26%)	249/639 (39%)	1·60[b]	1·27, 2·01	0.0001
Care joined up: no. reporting "almost always"/ total no. (%)[a]	196/629 (31%)	252/637 (40%)	1·34[b]	1·03, 1·74	0.0305
Overall satisfaction: no. reporting "very satisfied" / total no. (%)[a]	238/634 (38%)	359/648 (55%)	1·62[b]	1·30, 2·03	<0·0001

[a] Ordinal variable, dichotomized for ease of presentation. Full details of question and responses available in Table S6·
[b] Adjusted odds ratio from multi-level ordinal logistic regression. Adjusted by centre, baseline outcome score, practice list size and deprivation score. Practice is included as a random effect.
[c]Beta-coefficients. Analyses are multi-level linear regression models adjusted by centre, baseline outcome score, practice list size and deprivation score. Practice is included as a random effect.
[d] Use of the ©MMAS is protected by US Copyright laws. Permission for use is required. A licence agreement is available from Donald E Morisky, MMAS Research LLC 14725 NE 20th St Bellevue WA 98007 or from dmorisky@gmail.com

Appendix Table 6 Secondary outcomes at 9 and 15 months: full details of ordinal outcomes

	9 months		15 months	
**Outcome**	**Usual care (n=749)**	**Intervention (n=797)**	**Usual care (n=749)**	**Intervention (n=797)**
In general, would you say your health is:	N=666	N=672	N=631	N=642
Poor	142 (21%)	120 (18%)	137 (22%)	116 (18%)
Fair	287 (43%)	284 (42%)	264 (42%)	284 (44%)
Good	186 (28%)	205 (31%)	169 (27%)	177 (28%)
Very good	45 (7%)	58 (9%)	51 (8%)	58 (9%)
Excellent	6 (1%)	5 (1%)	10 (2%)	7 (1%)
When receiving care in the last six months did you discuss what was most important for you in managing your own health?	N=634	N=639	N=599	N=612
Not at all	109 (17%)	66 (10%)	110 (18%)	69 (11%)
Rarely	115 (18%)	86 (13%)	111 (19%)	69 (11%)
Some of the time	243 (38%)	238 (37%)	225 (38%)	218 (36%)
Almost always	167 (26%)	249 (39%)	153 (26%)	256 (42%)
Do you think the support and care you receive is joined up and working for you?	N=629	N=637	N=603	N=614
Not at all	91 (14%)	46 (7%)	77 (13%)	49 (8%)
Rarely	78 (12%)	55 (9%)	85 (14%)	49 (8%)
Some of the time	264 (42%)	284 (45%)	268 (44%)	259 (42%)
Almost always	196 (31%)	252 (40%)	173 (29%)	257 (42%)
In general, how satisfied are you with the care that you have had at your GP surgery or health centre?	N=634	N=648	N=608	N=614
Very dissatisfied	24 (4%)	13 (2%)	20 (3%)	12 (2%)
Fairly dissatisfied	40 (6%)	29 (4%)	31 (5%)	32 (5%)
Neither satisfied nor dissatisfied	81 (13%)	59 (9%)	91 (15%)	50 (8%)
Fairly satisfied	251 (40%)	188 (29%)	230 (38%)	175 (29%)
Very satisfied	238 (38%)	359 (55%)	236 (39%)	345 (56%)

Appendix Table 7 Completion of the 3D reviews

Outcome	Intervention (n=797) n/N (%)[a]
**Out of those who had at least one GP or Nurse review:**	
Patients most important problem noted[b]	616/622 (99%)
EQ5D pain question noted[b]	611/622 (98%)
Depression screening: PHQ9 entered[b]	599/622 (96%)
Patient agenda printed[b,d]	579/622 (93%)
Medication adherence noted[c]	506/599 (84%)
At least one patient goal noted[c]	590/599 (98%)
At least one patient action noted in health plan[c]	559/599 (93%)
At least one GP action noted in health plan[c]	554/599 (92%)
3D health plan printed[c]	461/598 (77%)

[a] Denominators related to the number of people eligible e.g. 622 patients had at least one nurse review and 599 had at least one GP review.
[b] In at least one nurse review
[c] In at least one GP review

Appendix Table 8· Complier averaged causal effect (CACE) analysis

	Mean EQ-5D-5L (SD), n at 15-month follow up		Adjusted difference in means	95% Confidence Interval	P-value
	Usual care	Intervention			
Participants by amount of intervention received:					
None (no GP and no nurse 3D appointments)	0·517 (0·311), 670	0·418 (0·336), 107			
Partial (at least one GP or nurse 3D appointment)		0·498 (0·336), 207	0·00[a]	-0·04, 0·03	0·796
Full (two GP and two nurse 3D appointments)		0·609 (0·256), 377	0·00[b]	-0·03, 0·02	0·798

All analyses are adjusted by centre, baseline EQ-5D-5L score, GP practice list size and GP practice deprivation score. GP practice is included as a cluster effect in the estimation of the variance-covariance matrix.
[a]Combining those in the none and partial compliance groups into the non-compliance group
[b]Combining those in the partial and full compliance group into compliance group

Notes:

Compliance (at the patient level) was defined as 'full' if two GP 3D appointments and two nurse 3D appointments were attended over 15 months; 'partial' – at least one GP or nurse 3D appointment attended, but not full attendance; and 'none' – no GP 3D appointment and no nurse 3D appointment attended.

Using an instrumental variable regression model with randomised group as the instrument and an indicator variable for compliance, the CACE analysis was conducted in two ways: first combining the partial and none compliers into the non-compliance group and, second, combining those in the partial and full compliance group into the compliance group. Both analyses show that there is no evidence of a difference in effect in the intervention group compared with the usual care group. Although there appears to be a trend of greater effect of the intervention in those who had full attendance, there was no difference between trial arms after adjustment because greater attendance was associated with higher EQ-5D-5L at baseline.

Appendix Updated systematic review incorporating previous trials and this trial

The last search for the Cochrane review of interventions for multimorbidity was conducted in September 2015.[9] We conducted searches in Medline and the Cochrane library in August 2017 using a search strategy adapted from that in the Cochrane review to identify trials published since September 2015, and attempted to update the meta-analyses in respect of quality of life and the PACIC measure in the light of these more recent trials and the 3D trial. This updated review identified a further 11 studies.[10-20] and one previously identified study with more recent published data.[21]

We have included the trial by Kennedy et al[22] in the above analyses because it was included in the Cochrane review, although is not described by the authors as an intervention for multimorbidity, and patients did not have to have multimorbidity to be included. Similarly several of the other trials are interventions for specific comorbid combinations of conditions, and not appropriate as a general approach to managing multimorbidity.

With respect to quality of life, the Cochrane review identified ten trials with relevant data.[22-31] These described studies which varied widely in terms of eligible population, setting and outcome measures. The Cochrane review authors were able to enter six of these studies into a meta-analysis, but did not report a pooled effect size due to substantial heterogeneity (I^2=73%). In our updated review we identified seven further trials reporting quality of life.[11,13-16,18,20,21] We have combined the results from the trials from the Cochrane review, the additional trials we identified and the results of the 3D trial and shown these in a Forest plot (see Appendix Figure 1). The data from the individual studies previously included in the Cochrane review are reported slightly differently in this figure from the data used in the original review because this figure is based on the generic inverse variance method which takes account of adjusted rather than unadjusted analyses of effect where these are available.

In extracting data for this analysis we chose any measure described by the authors as a measure of quality of life. Where studies used the SF36 we included data for the Physical Health summary score. Where studies reported data at multiple time-points we used the time-point closest to 12 months follow-up.

References for the studies within this figure are included in the bibliography as follows: Katon,[30] Martin,[21] Kennedy,[22] Boult[20] Barley,[27] Coventry,[29] Garvey,[28] Ekdahl,[18] Muth,[13] Koberlein-Neu,[15] Mercer,[14] Fortin,[16] Gonzalez Ortega,[11] Salisbury (refers to this paper).

Appendix Figure 1 Forest plot of trials of interventions in primary care for patients with multimorbidity

Study or Subgroup	Std. Mean Difference	SE	Intervention Total	Control Total	Weight	Std. Mean Difference IV, Random, 95% CI	Year
Katon 2010	0.3873	0.1481	94	92	3.3%	0.39 [0.10, 0.68]	2004
Kennedy 2013	0	0.023	1609	2382	22.4%	0.00 [-0.05, 0.05]	2013
Boult 2013	-0.0998	0.0667	485	419	11.0%	-0.10 [-0.23, 0.03]	2013
Barley 2014	-0.0067	0.2414	32	37	1.4%	-0.01 [-0.48, 0.47]	2014
Martin 2015	0.1529	0.2684	30	26	1.1%	0.15 [-0.37, 0.68]	2015
Coventry 2015	0.1305	0.1122	152	167	5.3%	0.13 [-0.09, 0.35]	2015
Garvey 2015	0.8154	0.315	22	22	0.8%	0.82 [0.20, 1.43]	2015
Ekdahl 2015	-0.0628	0.1186	167	124	4.9%	-0.06 [-0.30, 0.17]	2015
Muth 2016	-0.0233	0.2297	40	36	1.5%	-0.02 [-0.47, 0.43]	2016
Fortin 2016	0.2431	0.1131	155	160	5.3%	0.24 [0.02, 0.46]	2016
Koberlein-Neu 2016	-0.02	0.069	142	142	10.5%	-0.02 [-0.16, 0.12]	2016
Mercer 2016	0.14	0.125	67	67	4.5%	0.14 [-0.10, 0.38]	2016
Gonzalez-Ortega 2017	0.3485	0.1648	71	79	2.8%	0.35 [0.03, 0.67]	2017
Salisbury 2018	0	0.01	797	749	25.3%	0.00 [-0.02, 0.02]	2018
**Total (95% CI)**			**3863**	**4502**	**100.0%**	**0.04 [-0.02, 0.10]**	

Heterogeneity: $Tau^2 = 0.00$; $Chi^2 = 28.00$, df = 13 (P = 0.009); $I^2 = 54\%$
Test for overall effect: Z = 1.39 (P = 0.16)

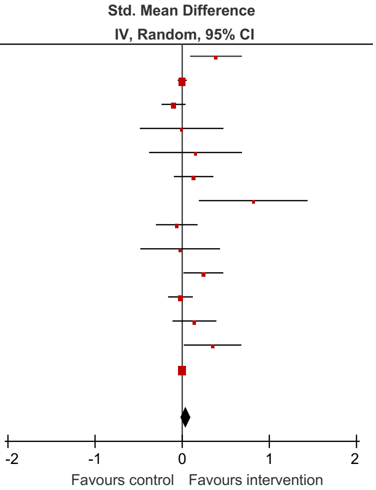

Figure 1 provides further evidence of little or no benefit in terms of quality of life, in that the pooled effect estimate is very small and the confidence interval overlaps zero. The updated analysis also shows high levels of heterogeneity so the pooled effect should be treated with considerable caution. There is also the possibility of publication bias, since a funnel plot shows asymmetry with the largest trials showing no evidence of effect (Figure 2).

Appendix Figure 2 Funnel plot

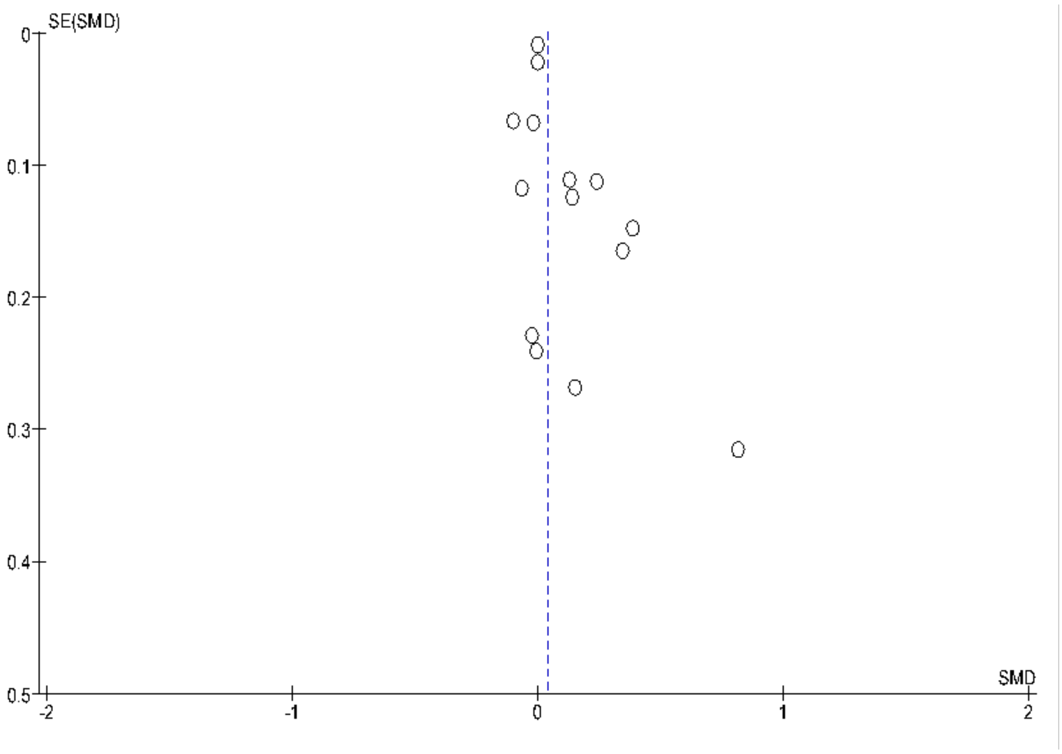

With regard to the PACIC measure, the Cochrane review identified two studies reporting this outcome.[29,32] We identified one more recent study.[12] The different studies reported data in different ways and were unsuitable for meta-analysis. However, all of the studies which have reported this outcome, including the 3D study, have confirmed that interventions to improve management of multimorbidity have a positive effect on patient-centred chronic care management as measured by PACIC.

References

1. Bayliss EA, Ellis JL, Steiner JF. Seniors' self-reported multimorbidity captured biopsychosocial factors not incorporated into two other data-based morbidity measures. *J Clin Epidemiol* 2009; **62**(5): 550-7.e1.
2. Zigmond AS, Snaith RP. The hospital anxiety and depression scale. *Acta Psychiatr Scand* 1983; **67**(6): 361-70.
3. Morisky DE, Ang A, Krousel-Wood M, Ward P. Predictive validity of a medication adherence measure in an outpatient setting. *J Clin Hypertens* 2008; **10**(5): 348-54.
4. Glasgow RE, Wagner EH, Schaefer J, Mahoney LD, Reid RJ, Greene SM. Development and validation of the Patient Assessment of Chronic Illness Care (PACIC). *Med Care* 2005; **43**(5): 436-44.
5. Mercer SW, Maxwell M, Heaney D, Watt GC. The consultation and relational empathy (CARE) measure: development and preliminary validation and reliability of an empathy-based consultation process measure. *Fam Pract* 2004; **21**(6): 699-705.
6. Bice TW, Boxerman SB. A quantitative measure of continuity of care. *Med Care* 1977; **15**(4): 347-9.
7. Garrison GM, Keuseman R, Bania B, Robelia P, Pecina J. Visit Entropy Associated with Hospital Readmission Rates. *Journal of the American Board of Family Medicine : JABFM* 2017; **30**(1): 63-70.
8. Reeves D, Campbell SM, Adams J, Shekelle PG, Kontopantelis E, Roland MO. Combining multiple indicators of clinical quality - An evaluation of different analytic approaches. *Med Care* 2007; **45**(6): 489-96.
9. Smith SM, Wallace E, O'Dowd T, Fortin M. Interventions for improving outcomes in patients with multimorbidity in primary care and community settings. *Cochrane Database of Systematic Reviews* 2016; (3).
10. Jager C, Freund T, Steinhauser J, et al. Impact of a tailored program on the implementation of evidence-based recommendations for multimorbid patients with polypharmacy in primary care practices-results of a cluster-randomized controlled trial. *Implementation Science* 2017; **12**(1): 8.
11. Gonzalez-Ortega M, Gene-Badia J, Kostov B, Garcia-Valdecasas V, Perez-Martin C. Randomized trial to reduce emergency visits or hospital admissions using telephone coaching to complex patients. *Fam Pract* 2017; **34**(2): 219-26.
12. Fried TR, Niehoff KM, Street RL, et al. Effect of the Tool to Reduce Inappropriate Medications on Medication Communication and Deprescribing. *J Am Geriatr Soc* 2017; **14**: 14.
13. Muth C, Harder S, Uhlmann L, et al. Pilot study to test the feasibility of a trial design and complex intervention on PRIoritising MUltimedication in Multimorbidity in general practices (PRIMUMpilot). *Bmj Open* 2016; **6**(7): e011613.
14. Mercer SW, Fitzpatrick B, Guthrie B, et al. The CARE Plus study - a whole-system intervention to improve quality of life of primary care patients with multimorbidity in areas of high socioeconomic deprivation: exploratory cluster randomised controlled trial and cost-utility analysis. *BMC Medicine* 2016; **14**(1): 88.
15. Koberlein-Neu J, Mennemann H, Hamacher S, et al. Interprofessional Medication Management in Patients With Multiple Morbidities. *Deutsches Arzteblatt International* 2016; **113**(44): 741-8.
16. Fortin M, Chouinard MC, Dubois MF, et al. Integration of chronic disease prevention and management services into primary care: a pragmatic randomized controlled trial (PR1MaC). *CMAJ Open* 2016; **4**(4): E588-E98.
17. Damush TM, Kroenke K, Bair MJ, et al. Pain self-management training increases self-efficacy, self-management behaviours and pain and depression outcomes. *European Journal of Pain* 2016; **20**(7): 1070-8.
18. Ekdahl AW, Wirehn AB, Alwin J, et al. Costs and Effects of an Ambulatory Geriatric Unit (the AGe-FIT Study): A Randomized Controlled Trial. *J Am Med Dir Assoc* 2015; **16**(6): 497-503.
19. Edelman D, Dolor RJ, Coffman CJ, et al. Nurse-led behavioral management of diabetes and hypertension in community practices: a randomized trial. *J Gen Intern Med* 2015; **30**(5): 626-33.
20. Boult C, Leff B, Boyd CM, et al. A matched-pair cluster-randomized trial of guided care for high-risk older patients. *J Gen Intern Med* 2013; **28**(5): 612-21.
21. Martin PR, Aiello R, Gilson K, Meadows G, Milgrom J, Reece J. Cognitive behavior therapy for comorbid migraine and/or tension-type headache and major depressive disorder: An exploratory randomized controlled trial. *Behav Res Ther* 2015; **73**: 8-18.
22. Kennedy A, Bower P, Reeves D, et al. Implementation of self management support for long term conditions in routine primary care settings: cluster randomised controlled trial. *BMJ* 2013; **346**: f2882.
23. Sommers LS, Marton KI, Barbaccia JC, Randolph J. Physician, nurse, and social worker collaboration in primary care for chronically ill seniors. *Arch Intern Med* 2000; **160**(12): 1825-33.
24. Morgan MA, Coates MJ, Dunbar JA, Reddy P, Schlicht K, Fuller J. The TrueBlue model of collaborative care using practice nurses as case managers for depression alongside diabetes or heart disease: a randomised trial. *Bmj Open* 2013; **3**(1).

25. Krska J, Cromarty JA, Arris F, et al. Pharmacist-led medication review in patients over 65: a randomized, controlled trial in primary care. *Age Ageing* 2001; **30**(3): 205-11.
26. Hogg W, Lemelin J, Dahrouge S, et al. Randomized controlled trial of anticipatory and preventive multidisciplinary team care: for complex patients in a community-based primary care setting. *Can Fam Physician* 2009; **55**(12): e76-85.
27. Barley EA, Walters P, Haddad M, et al. The UPBEAT nurse-delivered personalized care intervention for people with coronary heart disease who report current chest pain and depression: a randomised controlled pilot study. *PLoS One* 2014; **9**(6): e98704.
28. Garvey J, Connolly D, Bol, F., Smith S. OPTIMAL, an occupational therapy led self-management support programme for people with multimorbidity in primary care: a randomized controlled trial. *BMC family practice* 2015; **16**: 59.
29. Coventry P, Lovell K, Dickens C, et al. Integrated primary care for patients with mental and physical multimorbidity: cluster randomised controlled trial of collaborative care for patients with depression comorbid with diabetes or cardiovascular disease. *BMJ* 2015; **350**(h638).
30. Katon WJ, Lin EH, Von KM, et al. Collaborative care for patients with depression and chronic illnesses. *N Engl J Med* 2010; **363**(27): 2611-20.
31. Martin PR, Aiello R, Gilson K, Meadows G, Milgrom J, Reece J. Co-morbid recurrent headaches and major depressive disorder: An RCT of cognitive behavior therapy. 2013 International Headache Congress of the International Headache Society and American Headache Society; 2013: Boston, MA United States; 2013.
32. Boult C, Reider L, Frey K, et al. Early effects of "Guided Care" on the quality of health care for multimorbid older persons: a cluster-randomized controlled trial. *J Gerontol A Biol Sci Med Sci* 2008; **63**(3): 321-7.
